# Nephron-sparing management of Xanthogranulomatous pyelonephritis presenting as spontaneous renal hemorrhage: a case report and literature review

**DOI:** 10.1186/s12894-018-0354-3

**Published:** 2018-06-05

**Authors:** William Keith Ballentine, Fernandino Vilson, Raymond B Dyer, Majid Mirzazadeh

**Affiliations:** 1 Department of Urology, Wake Forest Baptist Health, Medical Center Blvd, Winston-Salem, NC 27157 USA; 2 Wake Forest School of Medicine, Winston-Salem, NC 27157 USA; 3 Department of Radiology, Wake Forest Baptist Health, Winston-Salem, NC 27157 USA

**Keywords:** Xanthogranulomatous pyelonephritis, Spontaneous renal hemorrhage, Wunderlich syndrome, Subcapsular renal hematoma, Case report

## Abstract

**Background:**

Xanthogranulomatous pyelonephritis (XGP) is an uncommon infectious disease of the kidney known to mimic other renal maladies. A rare presentation of this uncommon disease is spontaneous renal hemorrhage (SRH).

**Case presentation:**

We report a case of XGP in a 58 year old woman who presented with abdominal pain, hematuria, and radiating left flank pain. CT scan was felt to be consistent with perirenal hemorrhage abutting a fat-containing renal mass. The patient was eventually taken to surgery for left partial nephrectomy. Pathology report returned as XGP, and the patient has no complications from this disease process at 8 month follow up.

**Conclusion:**

Our search of the literature shows XGP presenting as SRH to be a rare clinical entity. Furthermore, this is the first such case managed with a nephron-sparing approach. The “great imitator” XGP should be added to the differential for patients presenting with spontaneous renal hemorrhage.

## Background

XGP is an uncommon infectious disease of the kidney first described in 1918 by Schlagenhaufer and characterized by chronic obstruction and inflammation [1]. The chronic inflammatory renal mass invades the renal parenchyma, replacing it with lipid laden macrophages. Pathologically, the lipid laden macrophages give the renal parenchyma a “tan-yellow” appearance [2]. SRH is defined as a non-traumatic, spontaneous renal bleed into the subcapsular and/or perirenal space. It was first observed by Bonet in 1679 and further described by Wunderlich in 1856 [3, 4]. This is only the 5th case report of XGP presenting as SRH.

Herein, we reported a female presenting with abdominal pain and hematuria with radiographic findings consistent with SRH who was found to have XGP as the underlying cause of her illness. We also review the epidemiology, diagnosis, and management of these two conditions.

## Case presentation

**Fig. 1:**
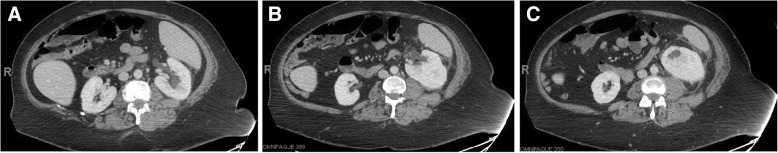
Axial CT scan demonstrating left renal hydronephrosis **a** and a non enhancing lesion of the left kidney **c** (arrowhead). Asymmetric psosas muscle **b** is identified and noticeable fat stranding along the posterior peritoneal wall (arrowhead). Ureteral calyces identified and demostrate distention possibly hemorrhaging

**Fig. 2:**
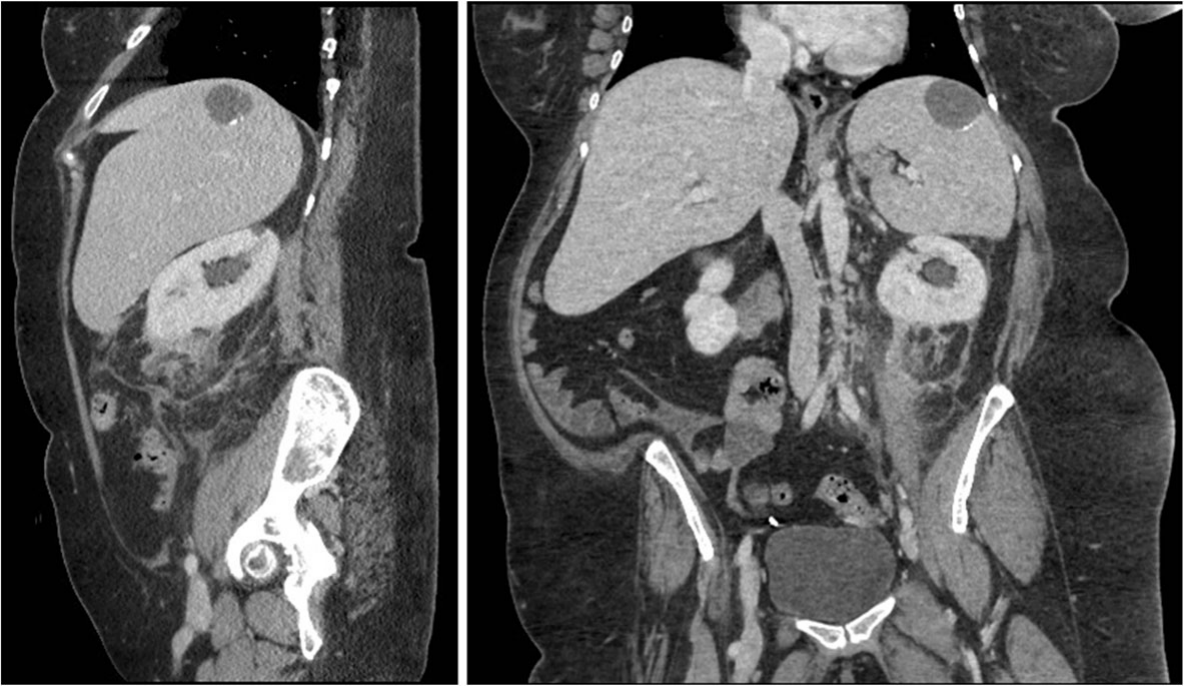
Coronal and sagittal CT scan demonstrating abnormal mass of left kidney, fat stranding, and hepatospenomegaly. There is a 3.5 cm round low attenuated area with peripheral calcifications in the spleen. Likely relates to prior trauma or infection

A 58 year old female presented with hematuria and left flank pain that radiated to the abdomen. Computed tomography (CT) scan demonstrated a heterogeneous 8.3 X 6.5 X 4.9 cm fat-containing mass arising from the lower pole of the left kidney (Fig. 1c). CT also demonstrated an asymmetrically enlarged left psoas muscle (Fig. 1b), soft tissue stranding (Figs. 1a & 2), mild hydronephrosis, and splenomegaly (Fig. 2). Further evaluation of the CT scan goes on to reveal extensive fascial thickening involving the anterior and posterior left renal fascia. Based on these findings, hemorrhagic angiomyolipoma was felt to be the most likely condition. Past medical history was significant for bipolar disorder and chronic right foot wounds associated with contiguous spread of chronic osteomyelitis of the 5th metatarsal.

Physical exam was remarkable for enlarged body habitus (BMI 34.7), limited ROM in right shoulder, arm, and neck. Laboratory abnormalities included an elevated glucose (186 mg/dl) and calcium (6.9 mg/dl). White cell count, BUN, and creatinine levels were within normal limits. Urine culture demonstrated no growth in 18–24 h and showed < 10,000 col./ml.

The decision was made to treat the patient with open left partial nephrectomy. Intraoperatively, the mass was easily found along the inferior portion of the kidney. The mass had a large desmoplastic reaction surround it from a prior bleeding episode. Adhesions surrounding the vascular pedicle were released. The pedicle was then clamped and cold ischemia was induced after mannitol injection. Resection of the mass was performed by scoring the mass with Bovie electrocautery and carefully dissecting the mass from the normal parenchyma by blunt dissection with a Penfield nerve retractor. All bleeding vessels were ligated. There was 36 min of cold ischemia time and 200 ml of estimated blood loss. After appropriate closure of the surgical sites, the mass was sent to pathology and because of considerable need for pyelocaliceal closure a double J ureteral stent and foley were placed. Postoperatively, the patient experienced urinary retention after foley catheter removal. Once stable, she was discharged back to skilled nursing facility with a catheter in place that was later removed.

At 10 day postoperative outpatient follow up, the patient was doing well and asymptomatic. BUN and creatinine levels were within normal limits but calcium remained low. Ureteral stent was easily removed during office cystoscopy. Eight months later the patient reported no signs of flank pain or hematuria and has remained at her pre-operative baseline.

## Discussion

XGP is well known for its capacity to mimic many other disorders, most notably renal neoplasms [5, 6]. Previously published case series have shown a higher incidence of disease in older women, patients obstructed from nephrolithiasis, and infection by *Escherichia coli* or *Proteus mirabilis*. [7–9]. XGP is classified into either the more prominent diffuse form or a focal entity [10]. It is further subdivided into 3 stages (nephric, perinephric, and paranephric) on the basis of the extent of inflammatory response. In part due to the rarity of XGP, pre-operative diagnosis is difficult with most cases diagnosed post-operatively on pathological examination. Clinical diagnosis is hampered by non-specific symptoms, and pre-operative radiologic imaging has been found to be of only moderate assistance in diagnosis [11]. While the classic “bear’s paw” sign is reasonably diagnostic of XGP, further radiologic specificity is limited by the heterogenous nature of the disease. One study of 11 patients with XGP found that 91% of patients demonstrated extrarenal extension of inflammatory changes and 82% had multiple dilated calyces and abnormal parenchyma, but 27% had focal fat deposits and a separate 27% had extensive retroperitoneal inflammation up to and including inflammation of the abdominal wall [12].

SRH, also known as Wunderlich Syndrome, is another uncommon urologic condition. The etiology of SRH is most frequently due to renal neoplasm but has also been known to arise due to vascular disease or infection [13]. While the infectious etiologies of SRH are known to include emphysematous pyelonephritis, to the best of our knowledge this is only the 5th case in the literature of XGP presenting with Wunderlich syndrome, although this is difficult to assess due to the many different terms applied to the condition [14–17]. SRH may classically present with “Lenk’s triad” (acute flank pain, abdominal tenderness, and symptoms of internal bleeding), but the disease is also known to present similarly to other abdominal conditions such as appendicitis or dissecting abdominal aortic aneurysm [13, 18]. Unlike XGP, CT imaging of SRH patients is able to identify 100% of patients suffering from the disease, though it lacks sensitivity for the etiology of SRH. Ultrasound has also been used for the detection of SRH but was not shown to be as reliable.

Three of 4 XGP cases presenting with Wunderlich syndrome are summarised and can be reviewed in Table 1. Canale et al., in a letter to the editor, presents a case of retroperitoneal hemorrhage with hemorrhagic shock [15]. Briefly, a 36 year old woman presented with right lower abdominal pain with palpable mass over right pelvic area. Laboratory findings included microcytic anemia and elevated white blood cell count (12,000 per mm^3^). Urinalysis (UA) was positive for leukocytes and pyuria. Urine culture was negative.

**Table 1:** Summary of XGP cases presenting as Wunderlich syndrome

Case	Presentation	CBC	UA	Culture (urine or site)	Surgery	Pathology
Canle et al. [15], 2007	Right lower abdominal pain and asthenia	Hgb 8 9/dl WBC 12000/mm^3^	+leukocyte +Pyuria	*Streptococcus anginosus*	Right total nephrectomy	Foamy macrophages aggregates, neutrophils, fibrosis. Inflammatory cell infiltrate
Altinoluk et al. [17], 2012	Right flank pain, fever, nausea	Hgb 6.3 g/dl WBC 16.7/ uL	Hematuria only	*Proteus mirabilis*	Right total nephrectomy	Foamy, lipid laden macrophages, giant cells, granulomatous reaction and fibrosis
Sharma et al. [16], 2013	Left flank pain, intermittent fever, weakness x15days	Hgb 9 g/dl WBC 13500/mm3	–	*Klebsiella*	Left total nephrectomy	Chronic granulomatous pyelonephritis. With dilated vascular channels

CT scan demonstrated bilateral central staghorn calculi with replacement of renal parenchyma with low attenuated collections in a hydronephrotic pattern. CT also showed a lower pole mass in the right kidney consistent with perinephric inflammatory changes. T1 and T2 weighted MRI images of the mass showed multiple enhancing septa.

Two days following the initial presentation the patient was seen again for acute abdominal pain with a red cell count of 6 g/dl, blood pressure of 80/60, and was transferred to the intensive care unit for hemorrhagic shock. CT scan now showed an interval increase in the size of the renal mass and hemoperitoneum. After right nephrectomy pathological examination of the mass revealed abundant foamy macrophage aggregates, neutrophils, fibrosis, and inflammatory cell infiltrations, confirming the diagnosis of XGP [15].

Altinoluk et al., describe a case of XGP with spontaneous kidney rupture in a young female [16]. A 25 year old woman presented with sudden onset right flank pain associated with fever and nausea. She was previously treated for UTI the week prior. Her blood pressure was 90/55 mmHg, hemoglobin 6.3 g/dl, white blood cell count was 16,700 per mm^3^. UA revealed hematuria only. Abdominal ultrasound and computed tomography displayed a large hypoechoic mass (12 X 7 cm) around the right kidney which extended into the pelvis and paravertebral space. Exploration of the mass revealed a large perirenal hematoma, abscess, and renal rupture. Culture of the abscess grew Proteus mirabilis. Histopathological examination following right nephrectomy revealed foamy, lipid laden macrophages, giant cells, polymorphonuclear cells, granulomatous reaction and fibrosis. A diagnosis of XGP was made [16].

Lastly, Sharma et al., reported a case of a 60 year old male who presented with left sided flank pain, intermittent fever, and weakness for 15 days [17]. A known diabetic, the patient was anemic (Hgb 9.0) with an elevated white blood cell count (13,500 per mm^3^). Urine culture was positive for *Klebsiella* and the patient was subsequently started on IV antibiotics. MRI abdomen demonstrated a hydronephrotic left kidney with an ill-defined mass of the lower pole suggestive of perinephric hematoma. Further, the patient was suspected to have a hemorrhagic angiomyolipoma and was thus surgically explored. During exploration, the kidney was noted to be hydronephrotic with thinned out parenchyma and palpable thickening of the lower pole which warranted nephrectomy. Unlike the previous cases, lipid-laden macrophage aggregates were not seen though further histopathological examination of the mass revealed chronic granulomatous pyelonephritis with dilated vascular channels and no evidence of neoplasia [17].

### Management

The traditional approach to XGP has been radical nephrectomy, though a nephron-sparing approach has been reported in the management of focal cases of XGP [19–21]. Conservative management of XGP has been achieved with parenteral antibiotic therapy or a combination of oral and parenteral therapy which may be supplemented with drainage of the urinary tract and/or abscesses [22–24]. Conservative management has even proven successful in renal allograft patients with XGP [25]. However, conservative management is inappropriate for patients with stage III or diffuse XGP, as may be seen in many patients at the time of diagnosis. Nephron-sparing management has been successfully attempted in cases of multifocal XGP, a distinct entity from diffuse XGP. As XGP is often diagnosed first at pathological specimen, the decision for treatment is usually based on a presumptive diagnosis in which radical nephrectomy may be more appropriate.

Management of SRH is first pursued through conservative or minimally invasive approaches. As the underlying cause of SRH may be focally identified by renal arteriography, embolization is an important therapeutic treatment that may preclude the need for surgery. In cases of iatrogenic renal hemorrhage, the combined use of urokinase injections and external drainage has been reported as an effective strategy in patients with large hematomas who nonetheless had stable vital signs [26]. This approach could also prove useful in SRH though we can find no explicit report of this in the literature. In the case of the unstable patient, surgical exploration is necessary if angioembolization is not available or is unsuccessful. Surgical exploration can be especially important if the source of the bleeding is found to be a renal neoplasm. Even in cases where bleeding can be managed conservatively, surgical evacuation of the clot may be required in instances of hypertension secondary to Page kidney.

All four previous reports of XGP presenting with SRH were managed by radical nephrectomy. We report the first case of nephron-sparing surgery for XGP presenting with SRH. Our management decision was facilitated by a pre-operative differential heavily weighted towards AML as the etiology of SRH. Prior to this procedure our approach to cases involving a large XGP lesion would have been unlikely to include a nephron-sparing approach, though we are happy to report that such an approach is certainly technically feasible.

## Conclusion

This report describes a rare presentation of an uncommon infectious disease. Our review of the literature confirms the rarity of XGP presenting with SRH. The importance of a broad differential diagnosis for this condition cannot be overlooked. Nephron-sparing surgery should always be considered as a possibility even in these difficult cases.

## Abbreviations

CT: Computed tomography
Hgb: Hemoglobin
SRH: Spontaneous renal hemorrhage
UA: Urinalysis
WBC: White blood cell count
XGP: Xanthogranulomatous Pyelonephritis
